# Aerodynamics and three-dimensional effect of a translating bristled wing at low Reynolds numbers

**DOI:** 10.1038/s41598-022-18834-0

**Published:** 2022-09-02

**Authors:** Wenjie Liu, Mao Sun

**Affiliations:** Institute of Fluid Mechanics, Beihang University, Beijing, 100191 China

**Keywords:** Fluid dynamics, Biological physics

## Abstract

The smallest insects fly with bristled wings at very low Reynolds numbers (*Re*) and use the drag of the wings to provide the weight-supporting force and thrust. Previous studies used two-dimensional (2-D) models to study the aerodynamic force and the detailed flow field of the bristled wings, neglecting the three-dimensional (3-D) effect caused by the finite span. At high *Re*, the 3-D effect is known to decrease the aerodynamic force on a body, compared with the 2-D case. However, the bristled wing operates at very low *Re*, for which the 3-D effect is unknown. Here, a 3-D model of the bristled wing is constructed to numerically investigate the detailed flow field and the aerodynamic force of the wing. Our findings are as follows: The 3-D effect at low *Re* increases the drag of the bristled wing compared with that of the corresponding 2-D wing, which is contrary to that of the high-*Re* case. The drag increase is limited to the tip region of the bristles and could be explained by the increase of the flow velocity around the tip region. The spanwise length of the drag-increasing region (measuring from the wing tip) is about 0.23 chord length and does not vary as the wing aspect ratio increases. The amount of the drag increment in the tip region does not vary as the wing aspect ratio increases either, leading to the decrease of the drag coefficient with increasing aspect ratio.

## Introduction

Flying birds and insects generally have one or more pairs of wings that resemble flat plates or membranes. By flapping their wings, they can generate a vertical aerodynamic force sufficient to support their weight. However, when researchers set their eyes on the barely visible miniature insects with a wing length of less than 0.5 mm, they discovered a leaky ”bristled wing” that comprised a narrow membranous blade and an array of slender bristles, remarkably different from the impermeable membranous wing^1–3^. This configuration has not yet been observed in insects of larger sizes but is quite common in miniature ones^4–6^.

During the flight of the miniature insects, the Reynolds number (*Re*) of the flapping wing (based on the average chord length and the average velocity of the wing) is very small, only about 10 and less. It is speculated that the bristled wing is the evolutionary adaptation of insects to the low-Reynolds-number conditions. Thus far, plenty of experimental measurements and numerical calculations have been performed to explore the aerodynamic characteristics of the bristled wings. The aerodynamic force (or torque) of a bristled wing was found only slightly smaller than that of the membranous wing with the same outline during steady or unsteady translational^7–10^ or rotational^7,11^ motions. For instance, the bristled wing of a tiny beetle, *Paratuposa placentis*, could produce about 66–96% of the aerodynamic force of the corresponding membranous wing when performing steady rotational motions at various angles of attack and low Reynolds numbers^11^. As to why the majority of the miniature insects have evolved the bristled wing rather than the membranous wing, researchers have provided various arguments and evidence, mainly based on the benefits of the bristled configuration at low *Re*. For example, the bristles could dramatically reduce the drag required to fling two wings apart^4,5,12–14^, and the bristled wing could reduce the inertial power requirements during the flapping motion^15,16^. Engels et al.^17^, on the other hand, focused on the disadvantage of the bristled wing in terms of the aerodynamic efficiency at higher *Re* and suggested that larger insects must use membranous wings for an efficient production of flight forces.

Whereas there are abundant researches on the aerodynamics of the bristled wing, the detailed flow around bristles, which helps to reveal the low-Reynolds-number aerodynamic mechanisms, has been less explored. Previous studies on the detailed flow of the bristled wings are mainly numerical simulations based on the two-dimensional (2-D) model. They are summarized as follows: Jones et al.^4^ compared the flow fields of the bristled wing at different angles of attack and found that the vortices in the gap between bristles became more diffuse and extended farther with decreasing angle of attack so that bristled wings might act more like membranous wings at the small angle of attack. Lee et al.^18^ analyzed the unsteady flow field of the bristled wing and discovered that *Re* would affect the gap flow significantly: At low *Re*, the diffusion of the viscosity was strong, and thus the gap flow responded more swiftly to the unsteady movement. Lee and Kim^19^ studied the aerodynamic response of the bristled and flat (membranous) wings in gusty flow and concluded that the bristled wing could alleviate the fluctuation of the aerodynamic force due to the gap flow. Wu et al.^9^ examined the steady translational motion of the bristled and flat wings and indicated that the pressure difference contributed to most of the drag of the flat wing but only half of the drag of the bristled wing whose another half of the drag came from the surface friction because a thick viscous layer formed around every bristle in the Stokes flow. Wu et al.^10^ found that the bristled wing could produce a very large unsteady drag peak as the flat wing did in rapid acceleration motion and that the flow around each bristle was a creeping flow.

To our best knowledge, the detailed flow field of the three-dimensional (3-D) bristled wing has not been studied. Although some researches^15–17^ have used the 3-D bristled wing model to calculate the aerodynamic forces and power, they did not investigate the detailed flow fields. Considering that the aerodynamic effect of the bristled wing at low *Re* resembles that of a membranous wing with a moderate aspect ratio, the 3-D effect of the bristled wing could be prominent but it is overlooked in the 2-D model. It thus necessitates a thorough investigation of the 3-D bristled wing model. The analysis of the detailed 3-D flow field will be important to the understanding of the aerodynamic force mechanisms of the bristled wing.

In this paper, we studied the aerodynamics and the detailed flow field of the 3-D bristled wing. We constructed the 3-D model of the bristled wing and performed the numerical simulation of the wing in steady translational motion at the angle of attack of $90^{\circ }$. By comparing the aerodynamic force of the 3-D bristled wing with that of the 2-D bristled wing, we specified the 3-D effect caused by the finite span at low Reynolds numbers. Then, through the analysis of the detailed flow field, we explained the aerodynamic force mechanisms. Furthermore, we explored the effect of changing the aspect ratio of the 3-D bristled wing.

## Models and methods

### Bristled wing models and non-dimensional parameters

**Figure 1:**
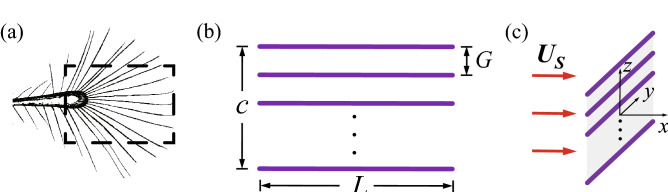
(**a**) Schematic diagram of the bristled wing of a fairyfly. (**b**) 3-D bristled wing model. (**c**) Flow condition and reference frame.

Figure 1a displays a schematic diagram of the bristled wing of a fairyfly, *Tinkerbella nana* (re-drawn from Ref. ^3^). During flight, the distal part of the wing, where the bristles are nearly parallel to the direction of the wing span, moves faster than the proximal part and generates most of the aerodynamic force. We thus based our 3-D bristled wing model on the distal part. In the 3-D bristled wing model (Fig. 1b), each bristle is represented by a circular cylinder with the diameter *d* and the length *L*. Both ends of the cylinder are smoothed using hemispheres. The cylinders are placed parallel to the wing span and the gap between neighboring cylinders is $G = 10d$, so the ratio of the gap to the diameter is $G/d = 10$ and it is consistent with the measured data in Refs. ^2,4,20^. Fifteen cylinders are arranged in total, numbered 1–15 from top to bottom, namely from the leading edge to the trailing edge of the wing. Cylinder No. 8 is the one at the mid-chord of the wing. In this configuration, the chord length of the wing is $c = 140d$. The aspect ratio (AR) of the wing is defined as the ratio of the wing span (the cylinder length *L*) to the chord length *c*. According to the micrographs of the real bristled wings in Ref. ^3^, we considered AR = 1.5–2.5 as biologically relevant. To examine the 3-D effect caused by the finite span, we also constructed a 2-D bristled wing model, which is a chordwise transection of the 3-D model and has been widely used by previous researchers (e.g., Refs. ^10,19^). Although the 3-D model includes the finite-span effect, it should be mentioned that this 3-D model is quite preliminary and not applicable to more complex cases. For example, the 3-D model assumes a uniform gap distance between adjacent bristles, while in a real bristled wing, the bristles are radially arranged.

According to the experimental observations of the flight of the miniature insects, the bristled wing would perform a deep U-shaped upstroke^21^, i.e., the “rowing” motion^22^, or two power strokes^15^. Both motions are characterized by a high velocity and a very large angle of attack, and produce most of the vertical aerodynamic force in a stroke cycle^15,21,22^. Therefore, in this study, we set the motion of the bristled wing as the steady translation at the angle of attack of $90^{\circ }$. Though a flapping wing is in rotational motion, here as a first step, we consider the simple and basic translational motion. Investigation of the translational motion would be enough to reveal the 3-D effect caused by the finite span. Rotational and flapping motions will be studied in our future work.

The flow field was solved in the object reference frame attached to the bristled wing, with its origin at the center of the wing, *y*-axis in the direction of the span, and *z*-axis in the direction of the chord (Fig. 1c). Viewed from the object reference frame, the uniform fluid flows in at the unit speed $U_S$ in the positive direction of the *x*-axis, perpendicular to the stationary wing. In this way, the whole flow field is steady, and symmetrical about the planes $y = 0$ and $z = 0$.

Because the bristles are so slender that the diameter of the bristle is two orders of magnitude smaller than the length of the bristle, it is necessary to define two Reynolds numbers. One is the characteristic parameter of the flow around the whole wing, namely $Re = \rho cU_S/\mu$, where $\rho$ and $\mu$ are the density and the dynamic viscosity coefficient of the fluid, respectively. The characteristic length is the chord length. The other is the characteristic parameter of the flow around one bristle, namely $Re_d = \rho dU_S/\mu$, the characteristic length of which is the diameter of the bristle. In this paper, *Re* is set to 10, and correspondingly, $Re_d$ is 0.0714. Both *Re* and $Re_d$ match the data given by Refs. ^12,15,20,23^.

The *x*-component of the resultant aerodynamic force exerted on the body is the drag *D* and can be divided into two parts: the surface friction $D_f$ and the surface pressure $D_p$. They are given by:

1 $$\begin{aligned} D_f= & {} \mathbf {x}\cdot 2\mu \int \mathbf {S}\cdot \mathbf {n} \mathrm {d} S, \end{aligned}$$

2 $$\begin{aligned} D_p= & {} \mathbf {x}\cdot \int -p\mathbf {n} \mathrm {d} S, \end{aligned}$$

where $\mathbf {x} = (1, 0, 0)$ is the unit vector in the *x*-direction, $\mathbf {n}$ the local outward unit vector normal to the body surface *S*, and $\mathbf {S}$ the strain rate tensor (both integrations are calculated on the body surface). For the 3-D bristled wing and the 2-D bristled wing, the drag coefficients are defined as $C_D = D/(0.5\rho U_{S}^{2}cL)$, and $C_D = D/(0.5\rho U_{S}^{2}c)$, respectively.

### Numerical method and grid test

In this study, OpenFOAM, an open-source software based on the finite volume method, was used to calculate the steady flow field and the aerodynamic force of the bristled wing. The governing equations of the flow are the steady-state incompressible Navier-Stokes equations. The velocity of the incoming flow $U_S$, the chord length *c*, and the density of the fluid $\rho$ are chosen as the scaling parameters to non-dimensionalize the equations. The dimensionless forms of the equations are

3 $$\begin{aligned}&\nabla \cdot \mathbf {u} = 0, \end{aligned}$$

4 $$\begin{aligned}&\mathbf {u}\cdot \nabla \mathbf {u}=-\nabla p+(1/Re)\nabla ^2\mathbf {u}, \end{aligned}$$

where $\mathbf {u}$ is the non-dimensional velocity, *p* the non-dimensional pressure, *Re* the Reynolds number, $\nabla$ the gradient operator, and $\nabla ^2$ the Laplacian operator. The equations are integrated and discretized on the controlling volume of each grid cell. Overall, the discretization schemes are second-order accurate. The interpolation scheme is Gauss linear. The surface normal gradient discretization uses the corrected method because of the mesh non-orthogonality. The convective term uses the bounded Gauss linearUpwind scheme, and the gradient term uses the Gauss linear scheme. Subsequently, simpleFoam, the steady-state solver based on the SIMPLE (Semi-Implicit Method for Pressure-Linked Equations) algorithm, was used to solve the velocity and pressure fields iteratively. The implementation of one iteration in simpleFoam is briefly described as follows: First, the pressure equation is solved using the velocity field specified initially or calculated from the last iteration step to obtain the new pressure field and correct the flux field. Secondly, the momentum corrector equation is solved using the new pressure field to obtain the new velocity field. Thirdly, evaluate the residual and go to the next iteration step till the convergence of the results.

**Figure 2:**
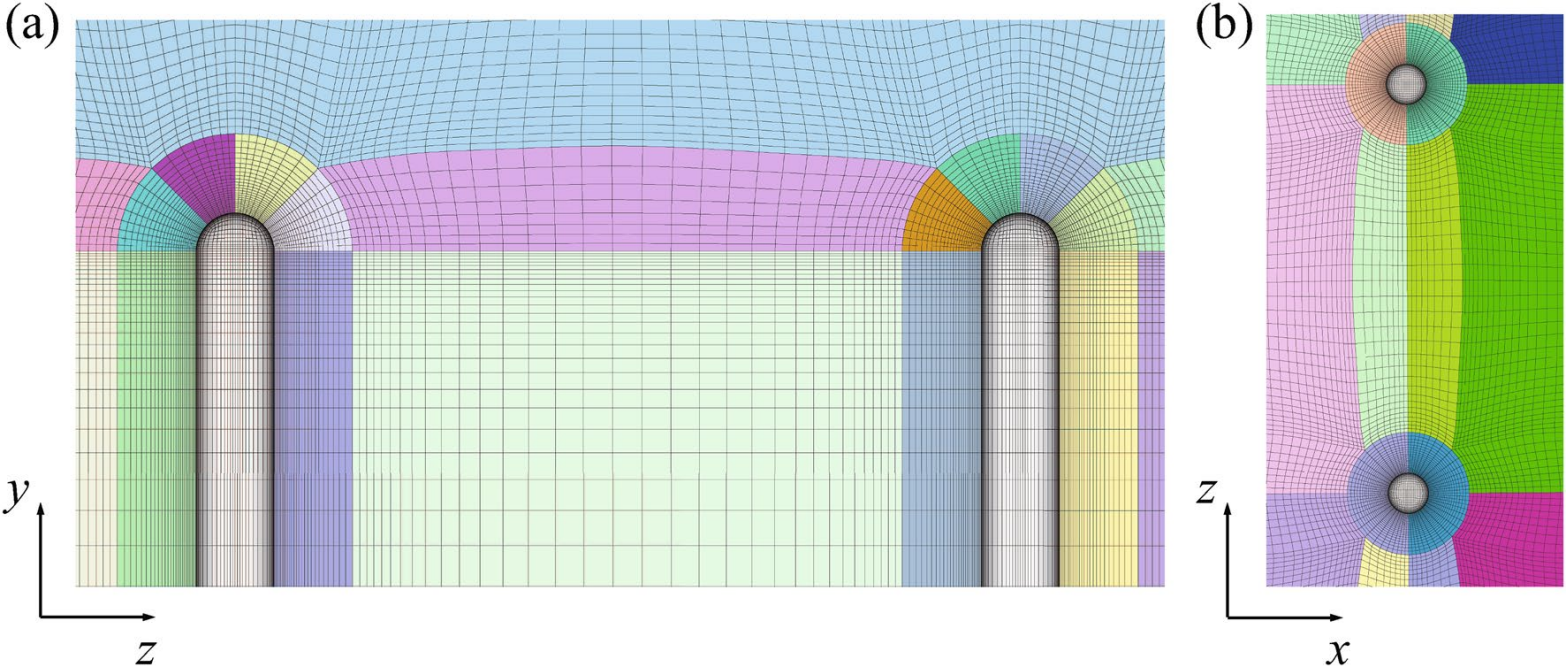
Near-field grid around the cylinders on (**a**) the plane $x = 0$, and (**b**) the plane $y = 0$.

A multi-block structured grid was generated for the 3-D bristled wing model. Figure 2 displays two sections of the near-field grid around the cylinders. The grid structure between every two cylinders is the same. The far-field boundary is 70*c* from the wing surface. As mentioned previously, the 2-D bristled wing model is a chordwise transection of the 3-D bristled wing model. Hence the grid structure of the 2-D wing is identical to that shown in Fig. 2b. Considering that the perturbation of the 2-D flow field propagates farther, the distance between the far-field boundary and the 2-D wing surface was extended to 140*c*.

As for the boundary conditions, at the wing surface, the normal gradient of the pressure, and the velocity are fixed to zero; at the far-field inflow boundary, the velocity is set to $U_S$ and the normal gradient of the pressure to zero; at the far-field outflow boundary, the normal gradient of the velocity is set to zero and the pressure to the reference pressure, $p_{ref}$. Additionally, as the whole flow field is symmetrical about the planes $y = 0$ and $z = 0$, only 1/4 of the grid needs to be calculated, and the symmetrical conditions are applied to these two planes.

To ensure that the simulation results are independent of the grid resolution, a coarse grid, a medium grid, and a fine grid (the numbers of cells of which are $1.45\times 10^6$, $5.54\times 10^6$, and $2.33\times 10^7$, respectively, in about $1.5^3$-fold increments) were generated for the 3-D bristled wing. The drag coefficients calculated using these three grids are 3.002, 2.998, and 3.001, respectively, confirming that the numerical results are almost independent of the computational grid. As the time consumed by the calculation of the fine grid is six times more than that of the medium grid, we chose the medium grid ultimately and generated other grids with the same arrangement of nodes as the medium grid. In the medium grid, 64 nodes are distributed around the circumference of each cylinder.

## Results and discussion

### Method validation

To validate the solver in calculating the flow field and the force distribution of a slender body at low Reynolds numbers, we compared the numerical results of two slender bodies in the steady flow with their corresponding analytical solutions. The analytical solutions approximate the physical realities at low Reynolds numbers in both cases.

The first case is the steady flow past a 2-D elliptic cylinder. The analytical solution based on Oseen’s approximation is given by Ref. ^24^. In this case, the Reynolds number based on the length of the major axis of the ellipse 2*a* is 0.2, and the incoming flow at the unit velocity in the positive direction of the *x*-axis is perpendicular to the major axis of the ellipse. The equation of the ellipse is

5 $$\begin{aligned} \frac{x^2}{b^2}+\frac{z^2}{a^2}=1, \end{aligned}$$

where $a = 70$, $b = 5.9161$, meaning that the ratio of the length of the major axis to the radius of the curvature at either end of the major axis (of the ellipse) equates to the ratio of the chord length to the bristle radius (of the bristled wing). The half focal length of the ellipse is $c = (a^2-b^2)^{1/2}$. Let us introduce the elliptical coordinates $(\xi , \eta )$ defined by:

6 $$\begin{aligned} x= & {} c~sh\xi cos \eta , \end{aligned}$$

7 $$\begin{aligned} z= & {} c~ch \xi sin \eta . \end{aligned}$$

**Figure 3:**
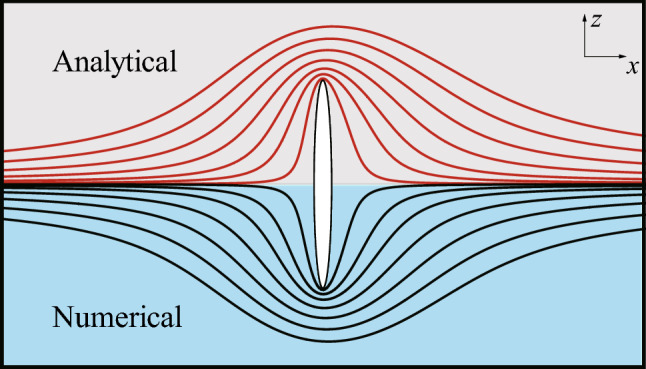
Analytical solution and numerical result of the streamline of the flow around the 2-D elliptic cylinder.

**Figure 4:**
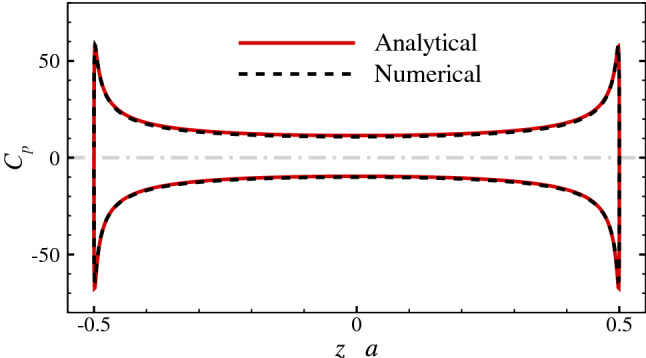
Analytical solution and numerical result of the pressure coefficient distribution along the major axis of the 2-D elliptic cylinder.

Defining the surface pressure coefficient as $C_p = (p-p_{\infty })/(0.5\rho U_{S}^{2})$, where $p_{\infty }$ is the pressure at infinity, the analytical solution of $C_p$ on the surface of the 2-D elliptic cylinder is given by:

$$\begin{aligned} C_p \approx (sh^2 \xi _0 + cos^2 \eta )\sum _{n=0}^{2} \alpha _n \big [e^{-(n-1)\xi _0} cos (n+1)\eta +e^{-(n+1)\xi _0} cos (n-1)\eta \big ], \end{aligned}$$

where $\xi _0 = 0.0847, \alpha _0 = -5.2709, \alpha _1 = 0.5144, \alpha _2 = -0.0064$. The analytical solution of the stream function is quite complicated, so its expression is omitted here. The comparisons between the analytical solutions and the numerical results of the streamline (Fig. 3) and the distribution of $C_p$ along the major axis (Fig. 4) both show great agreement.

**Figure 5:**
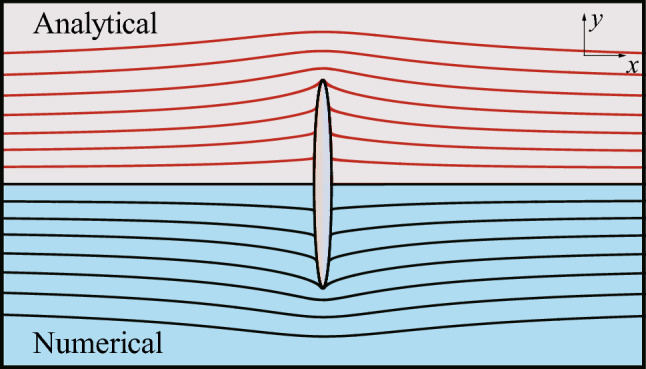
Analytical solution and numerical result of the streamline of the flow around the prolate spheroid in the longitudinal section.

**Figure 6:**
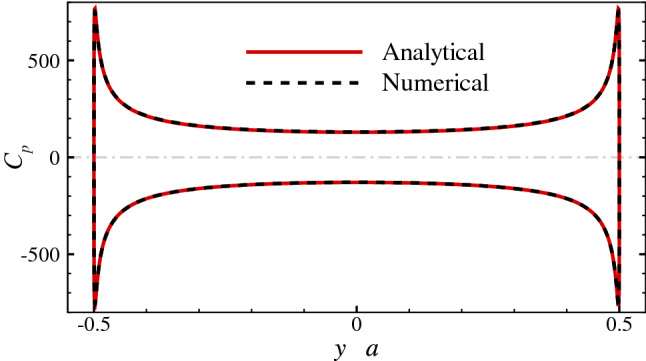
Analytical solution and numerical result of the pressure coefficient distribution along the major axis of the prolate spheroid.

The second case is the uniform flow past a prolate spheroid. The analytical solution based on Stokes’ approximation is given by Ref. ^25^. The Reynolds number of this case is the same as the first case, i.e., $Re = 0.2$. The incoming flow at the unit velocity in the positive direction of the *x*-axis is perpendicular to the major axis of the spheroid. The equation of the longitudinal section of the spheroid (within the plane $z = 0$) is

8 $$\begin{aligned} \frac{x^2}{b^2}+\frac{y^2}{a^2}=1, \end{aligned}$$

representing an ellipse of the same shape as the one in the first case. The analytical solution of the pressure coefficient at (*x*, *y*, 0) is given by:

9 $$\begin{aligned} C_p=-\frac{8a}{Re} \int _{-c}^{c}\alpha \frac{x}{(x^2+(y-\xi )^2)^{3/2}} \mathrm {d} \xi , \end{aligned}$$

where $\alpha = 0.1369$. The expression of the analytical solution of the velocity field is not given here. The comparisons between the analytical solutions and the numerical results of the streamline (Fig. 5) and the distribution of $C_p$ along the major axis (Fig. 6) both show excellent coincidence.

These two cases suggest that the solver used in this study is reliable. If comparing the distributions of the surface pressure coefficients in the two cases, we can find that both curves have the similar form, indicating that the flow at low Reynolds numbers will exert a huge force peak on the tips of the slender bodies. Furthermore, because the shapes of the ellipses are the same and the Reynolds numbers are equal in two cases, we can readily discern the different patterns of the flow bypassing a 3-D object and a 2-D object. For the 3-D object, the fluid can ”take the short-cut” around, and the streamline is relatively flat, while for the 2-D object, the flow is constrained to the plane, and the fluid can only ”squeeze” around the tips of the object, so the streamline is more tortuous.

### Drag of the bristled wing

**Figure 7:**
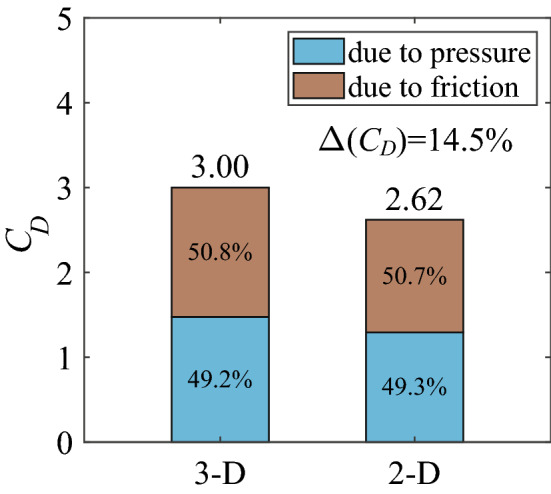
Drag coefficients of the 3-D and 2-D bristled wings. $\Delta (C_D)$ is the percentage of the increased drag coefficient of the 3-D case over the 2-D case.

In this section, we calculate the drag coefficient of the 3-D bristled wing with AR = 1.5 at $Re = 10$, and compare it with that of the 2-D bristled wing (cases of other aspect ratios are analyzed in a later section). The results are shown in Fig. 7, from which we can see that the drag coefficient of the 3-D bristled wing is greater than that of the 2-D bristled wing. This is contrary to the 3-D effect at high Reynolds numbers. For example, experimental studies of the flow around cylinders of finite length at the Reynolds number of $O(10^4)$ or higher showed that the drag coefficient increased with the ratio of length to diameter (aspect ratio)^26,27^, i.e., the drag coefficient of the 2-D cylinder is always greater than that of the 3-D cylinder. This is because the flow near the ends of the 3-D cylinder will turn and reach the low-pressure region in the leeward side, increasing the base pressure and reducing the pressure difference between the leeward and the windward^26^. Since the pressure difference contributes to almost all of the drag in high-Reynolds-number situations, the drag coefficient of the 3-D cylinder is thus reduced. We also performed numerical simulations of the 3-D plate wing with AR = 1.5 and the 2-D plate wing at chord-based $Re = 1000$; the calculated drag coefficient of the 2-D case is more than twice as large as that of the 3-D case, which further demonstrates that the 3-D effect at high Reynolds numbers will decrease the drag exerted on a bluff body. Our above results of the bristled wing, however, suggest a different mechanism of the 3-D effect at low Reynolds numbers that increases the drag coefficient of a body. For convenience, $\Delta (C_D)$ is used to represent the percentage of the increased drag coefficient of the 3-D case over the corresponding 2-D case. Here, $\Delta (C_D)$ of the whole bristled wing is 14.5%. In addition, we observe that the total drag is contributed approximately half by the surface pressure and half by the surface friction for both the 3-D and 2-D bristled wings (Fig. 7).

**Figure 8:**
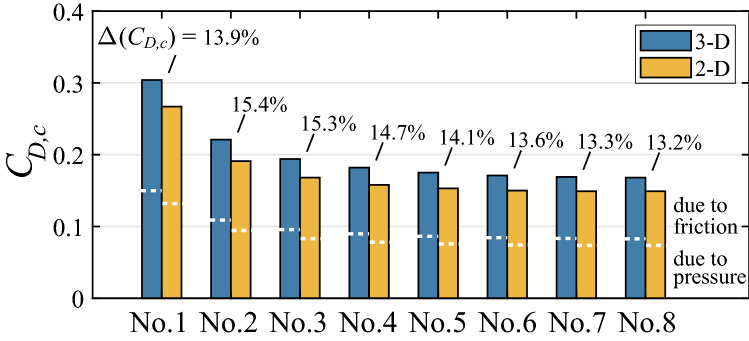
Drag coefficient of each cylinder in the 3-D and 2-D bristled wings. $\Delta (C_{D,c})$ is the percentage of the increased drag coefficient of the 3-D cylinder over the corresponding 2-D cylinder. White dotted lines separate the contributions of the surface pressure and friction to the drag of each cylinder.

Unlike the continuous wing, the bristled wing is constituted by an array of slender cylinders (Fig. 1b) and its drag is the sum of the drag of the cylinders. Therefore, let us first examine the drag of each cylinder (denoted by $D_c$). For each individual cylinder in the 3-D wing and the corresponding 2-D wing, the drag coefficients are defined as $C_{D,c} = D_c/(0.5\rho U_{S}^{2}cL)$ and $C_{D,c} = D_c/(0.5\rho U_{S}^{2}c)$, respectively. Note that the reference area used here is the same as that used in the non-dimensionalization of the drag of the bristled wing. $C_{D,c}$ of each cylinder in the 3-D and 2-D bristled wings is plotted in Fig. 8. Since the flow is symmetrical about the plane $z = 0$, only $C_{D,c}$’s of cylinders No. 1–8 are shown. $\Delta (C_{D,c})$ is used to represent the percentage of the increased drag coefficient of the 3-D cylinder over the corresponding 2-D cylinder. It can be seen that $\Delta (C_{D,c})$ of each cylinder is about 14% (between 13.2 and 15.4%). This is close to the value of $\Delta (C_D)$ of the whole bristled wing, 14.5%, indicating that the 3-D effect of every cylinder enhances the drag coefficient to a similar extent. From the figure, we also see that for both 3-D and 2-D cases, the drag coefficient of cylinder No. 1 is considerably larger than those of the inner cylinders, and the drag coefficients of cylinders No. 3–8 are almost the same. In other words, the drag is large on the leading edge and the trailing edge, and small but evenly-distributed in the inner chord of the bristled wing.

The white dotted lines in Fig. 8 separate the contributions of the surface pressure and friction to the drag of each cylinder, both of which are approximately 50%. This is a feature of the 2-D cylinder in the Stokes flow. We thus see that every slender 3-D cylinder of the bristled wing is locally in the Stokes flow of $Re_d = 0.0714$ although the bristled wing, as a whole, is in the flow of $Re = 10$.

**Figure 9:**
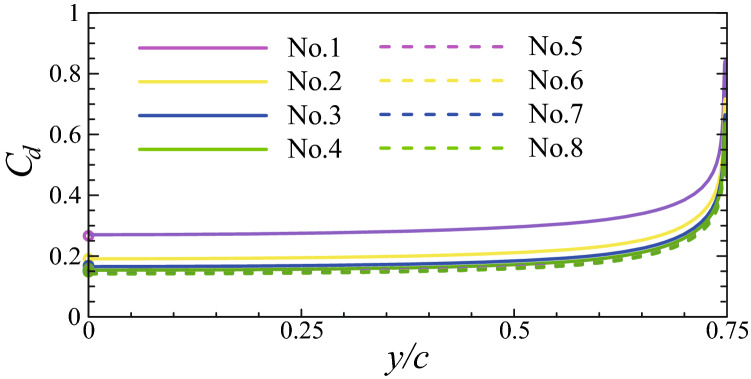
Spanwise distribution of the drag coefficient of each cylinder in the 3-D bristled wing. Circles on the vertical axis signify the drag coefficients of the corresponding cylinders in the 2-D bristled wing. Note that the abscissa is the spanwise position scaled by the chord length.

To find out where the increased drag coefficient of the 3-D cylinder comes from, let us look at the spanwise distribution of the drag of each cylinder. Denoting $C_d$ as the drag coefficient per unit length of the cylinder, the change of $C_d$ as a function of the spanwise position is plotted in Fig. 9 for each cylinder in the 3-D bristled wing. The distribution curves are drawn along only half of the span because the flow is symmetrical about the plane $y = 0$. The spanwise position is scaled by the chord length rather than the span here and throughout the paper; this is because the chord length remains unchanged when we investigate the effect of changing the aspect ratio in a later section. The values of the drag coefficients of the corresponding cylinders in the 2-D bristled wing are marked on the vertical axis with circles. What stands out in this figure is the resemblance of all curves: an almost horizontal line in the inner span and a sharp rise near the tip. From the mid-span to $y = 0.5c$ (2/3 of the wing span), $C_d$ of each 3-D cylinder is nearly the same as that of the corresponding 2-D cylinder; afterwards, it starts to grow; and when very close to the tip, it grows sharply, showing that $C_d$ at the cylinder tip is influenced by the 3-D effect. Therefore, the increased drag coefficients of the 3-D cylinders over the 2-D cylinders mainly come from the tip regions.

The results above have shown the following: (i) The drag coefficient of the 3-D bristled wing is higher than that of the 2-D bristled wing because the drag coefficient of each cylinder in the former is higher than that of the corresponding (2-D) cylinder in the latter. (ii) The increased drag coefficients of the 3-D cylinders over the 2-D cylinders mainly come from the tip regions. (iii) For both the 3-D and 2-D bristled wings, the drag coefficient is remarkably larger on the leading and trailing edges than in the inner chord of wing, and it is evenly-distributed in the inner chord. To reveal the underlying mechanisms of these aerodynamic force characteristics, we will investigate the flow field in the next section.

### Flow field and aerodynamic force mechanisms

#### The 2-D bristled wing

It has been demonstrated that the drag coefficient of the 3-D bristled wing, except near the tip regions, is approximately the same as that of the 2-D bristled wing. Therefore, let us first consider the 2-D bristled wing, the results of which will be helpful to the study of the 3-D bristled wing.

As the 2-D bristled wing is constituted by an array of cylinders, the multi-cylinder interaction may play an important role in the drag production of each cylinder, especially when the Reynolds number of the cylinder $Re_d$ is as low as 0.0714. At such a low Reynolds number, the viscous diffusion is very strong and affects a broad area around the cylinder. In order to examine the interaction between the cylinders in the bristled wing, the flow of a single 2-D cylinder at $Re_d$ is calculated for comparison.

The calculated drag coefficient of the single cylinder ($C_{D,c}$) is 0.519, which far exceeds those of the cylinders in the 2-D bristled wing: It is twice as large as that of the cylinder on the leading edge of the wing (see $C_{D,c}$ of cylinder No. 1 in Fig. 8), and is more than three times as large as those of the cylinders in the inner chord (see $C_{D,c}$’s of cylinders No. 3–8 in Fig. 8). This indicates that, although the gap between two adjacent cylinders of the wing is as large as 10*d*, the drag coefficient of each cylinder is considerably reduced due to the influence of other cylinders.

**Figure 10:**
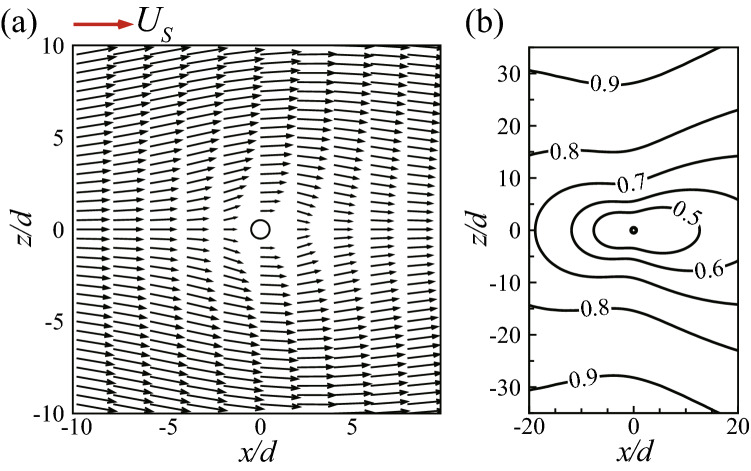
Flow field around a single 2-D cylinder at $Re_d = 0.0714$. (**a**) Velocity vectors in the near field. (**b**) Contours of the non-dimensional velocity magnitude (non-dimensionalized by $U_S$) in the farther field.

**Figure 11:**
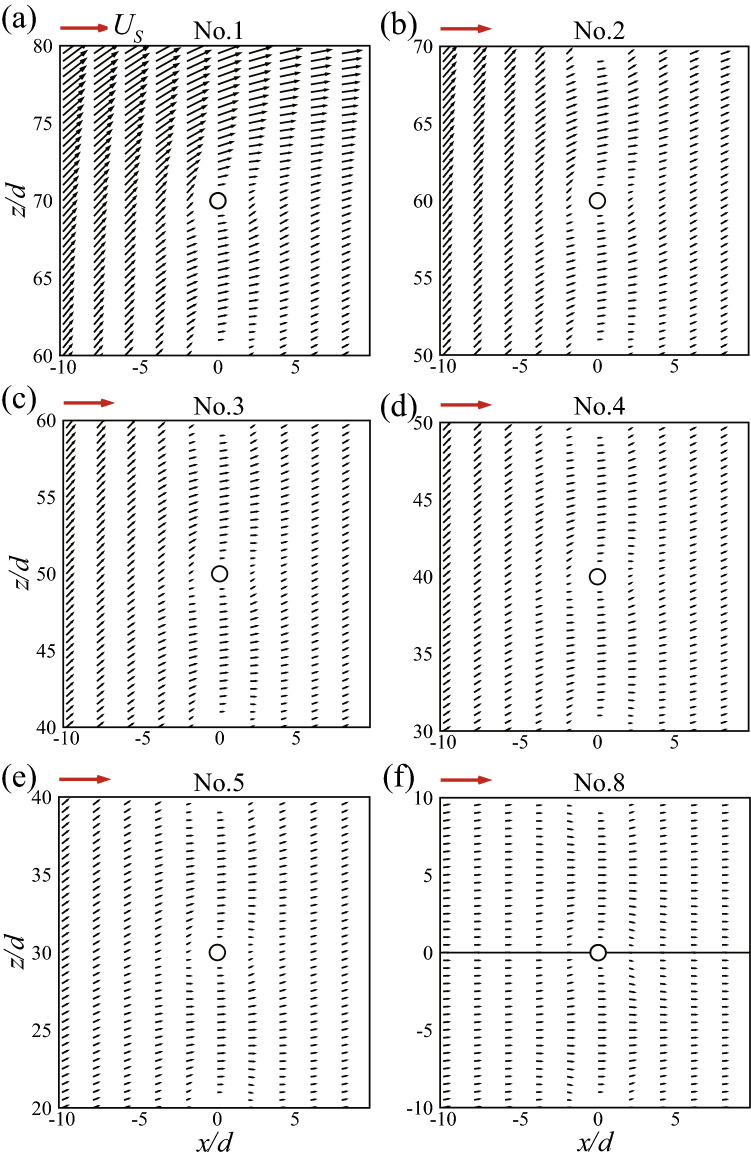
Flow fields around individual cylinders in the 2-D bristled wing. Cylinder No. 8 is in the mid-chord of the wing and the black line is but a mark of symmetry.

The flow field around the single cylinder is plotted in Fig. 10. We can see that the flow in the vicinity of the cylinder is greatly slowed down due to the strong viscosity. At 5*d* above or below the cylinder, the flow velocity is reduced from the free-stream velocity $U_S$ to merely about $0.5U_S$ (Fig. 10a), and even at 20*d*, still reduced to about $0.85U_S$ (Fig. 10b); only when the distance is larger than 20*d*, the reducing effect becomes relatively small and the flow velocity becomes fairly close to $U_S$. For a cylinder of the bristled wing, the flow around it is affected by more than one cylinder, so it is expected that the flow velocity will be reduced more severely. The flow fields around six cylinders of the wing are plotted in Fig. 11. As expected, the flow velocity around the cylinders of the wing is much lower than that around the single cylinder: The former is less than $0.2U_S$, except that around the cylinder at the leading edge, cylinder No. 1 (and the one at the trailing edge, cylinder No. 15, not shown in the figure). This explains why the drag coefficient of a cylinder in the bristled wing is much smaller than that of the single cylinder.

From Fig. 11, we can also see that the flow velocities around the two cylinders closest to the leading edge (cylinders No. 1 and 2) are higher than those around the rest; and the flow fields around cylinders No. 3, 4, 5, and 8 are almost identical, implying that the flow around a cylinder in the bristled wing is affected mainly by the two cylinders above and the two cylinders below it. Therefore, the flow velocities around different cylinders in the inner chord (i.e., cylinders No. 3–13), are reduced to the same degree, resulting in the equally low drag coefficients of the cylinders. However, for the leading edge of the wing (see cylinder No. 1 in Fig. 11), there is no cylinder above. Hence the flow velocity around is less reduced, and the drag coefficient is higher. The same is true for the trailing edge. This explains the chordwise distribution of the drag of the bristled wing.

#### The 3-D bristled wing

Now let us consider the aerodynamic force mechanism of the 3-D bristled wing. For the 2-D bristled wing, from the analysis in the last section, the mutual interaction of the cylinders has a strong effect on the flow field and the aerodynamic force. For the 3-D bristled wing, in addition to the interaction among the cylinders, there will be 3-D effect on the flow. In order to single out the 3-D effect, we first consider a single 3-D cylinder performing the same motion as the wing at $Re_d = 0.0714$. By comparing the flow of the 3-D single cylinder with that of the corresponding 2-D single cylinder, the 3-D effect can be identified.

**Figure 12:**
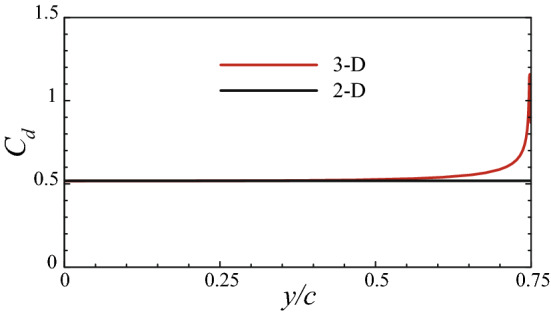
Spanwise distributions of the drag coefficients of the single 3-D and 2-D cylinders.

The model, the grid, the definitions of the non-dimensional drag coefficient ($C_{D,c}$) and the drag coefficient per unit span ($C_d$) of the single 3-D cylinder are the same as those of an individual cylinder in the 3-D bristled wing. The computed $C_{D,c}$ of the 3-D single cylinder is 0.538, larger than that of the 2-D single cylinder ($C_{D,c}$ = 0.519) by 3.66%. The spanwise distributions of the drag coefficients (i.e., $C_d$ varies as a function of *y*) of the 3-D and 2-D cylinders are plotted in Fig. 12 (again, due to symmetry, the distribution curves are drawn along only half of the span, as in Fig. 9). It is seen that $C_d$ of the 3-D cylinder is almost equal to that of the 2-D cylinder from the mid-span to $y = 0.6c$; and only in the tip region, it becomes larger than that of the 2-D cylinder. That is, the 3-D effect has a negligible influence on the drag of the inner span of the 3-D cylinder, and the increased drag coefficient over the 2-D cylinder comes from the tip regions of the 3-D cylinder.

**Figure 13:**
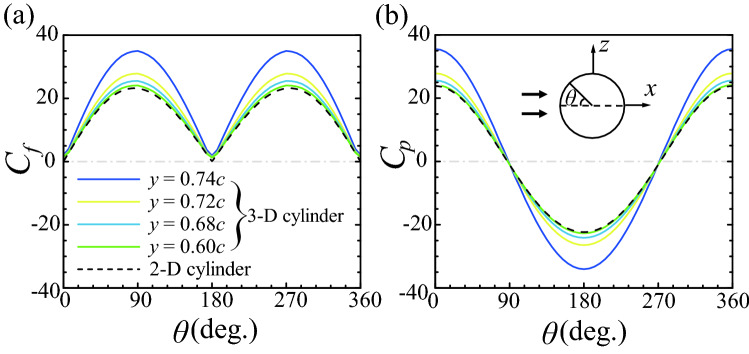
Circumferential distributions of (**a**) the surface friction coefficient, and (**b**) the surface pressure coefficient of the single 3-D and 2-D cylinders.

The drag on the cylinder is due to the frictional and pressure forces on the cylinder surface. Let us look at how these two contributions change in the tip region of the 3-D cylinder. The shear stress tangential to the surface is $\mathbf {f} = 2\mu [\mathbf {S}\cdot \mathbf {n} - (\mathbf {n}\cdot \mathbf {S}\cdot \mathbf {n})\mathbf {n}]$, so the magnitude of the shear stress along the circumference of the cylinder is $(\left| \mathbf {f}\right| ^2-\left| \mathbf {f}\cdot \mathbf {j}\right| ^2)^{0.5}$, where $\mathbf {j} = (0, 1, 0)$ is the unit vector in the *y*-direction. For a point on the cylinder surface, we define the surface friction coefficient as $C_f = (\left| \mathbf {f}\right| ^2-\left| \mathbf {f}\cdot \mathbf {j}\right| ^2)^{0.5}/(0.5\rho U_{S}^{2})$ and the surface pressure coefficient as $C_p = (p-p_{ref})/(0.5\rho U_{S}^{2})$, where the reference pressure $p_{ref}$ is the static pressure at the far-field outlet boundary. Figure 13 gives the circumferential distributions of the surface friction and pressure coefficients (i.e., $C_f$ and $C_p$ vary as functions of the circumferential angle $\theta$; $\theta = 0^{\circ }$ is at the windward side of the cylinder), at four spanwise positions in the tip region of the 3-D cylinder: $y = 0.60c, 0.68c, 0.72c$, and 0.74*c* (the distributions of $C_f$ and $C_p$ of the 2-D cylinder are also given in the figure for comparison). It can be seen that, at $y = 0.60c$, $C_f$ and $C_p$ are almost the same as their counterparts of the 2-D cylinder; it is even more so when $y < 0.60c$ (data not shown here). At $y = 0.68c$ and 0.72*c*, the magnitudes of $C_f$ and $C_p$ are slightly but noticeably larger than those of the 2-D cylinder, while at $y = 0.74c$, which is very close to the cylinder tip, $C_f$ and $C_p$ of the 3-D cylinder become much larger than those of the 2-D cylinder. It can also be observed in Fig. 13 that at each spanwise position, the magnitudes of $C_f$ and $C_p$ are approximately equal, i.e., in the tip region, the drag is still constituted approximately half by the frictional force and half by the pressure force. We thus see that it is the increase of the magnitudes of $C_f$ and $C_p$ near the cylinder tip that gives the increase of $C_d$ in the tip region.

From the above discussion, we have the following results: (i) The drag coefficient of the single 3-D cylinder is larger than that of the corresponding 2-D cylinder. (ii) The increment of the drag coefficient comes from the tip region of the 3-D cylinder. (iii) In the tip region, the surface friction and pressure are both increased, resulting in the increase of the drag there.

**Figure 14:**
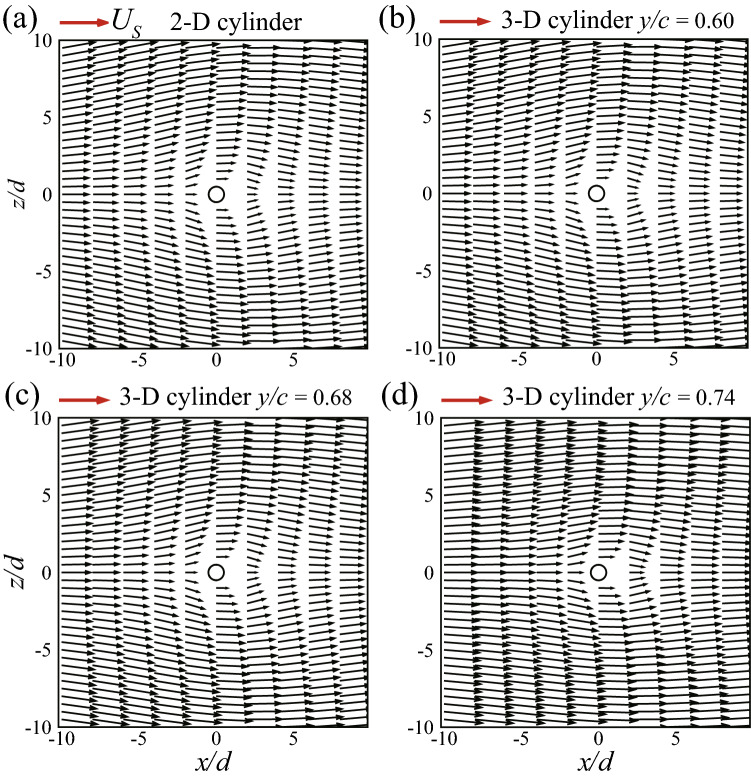
Flow fields in different sections of (**a**) the 2-D cylinder and (**b**–**d**) the 3-D cylinder.

Now, let us examine the flow field and explain why $C_f$ and $C_p$ are increased in the tip region of the 3-D cylinder. The sectional flow velocity vector plots in three sections in the tip region ($y = 0.60c, 0.68c$, and 0.74*c*) are given in Fig. 14 (the result of the 2-D cylinder is included for comparison). At the section of $y = 0.60c$, the flow velocity around the 3-D cylinder is almost the same as that of the 2-D cylinder (comparing Fig. 14b with Fig. 14a). At $y = 0.68c$, the flow velocity around the cylinder becomes larger (comparing Fig. 14c with Fig. 14a, b), and the shearing effects near the cylinder surface will be stronger. At $y = 0.74c$, which is very near the cylinder tip, the flow velocity is the largest (comparing Fig. 14d with Fig. 14a–c) and the shearing effects will be the strongest. It is known that in the Stokes flow, there is no inertial force, and that the pressure field variation and the frictional force are both due to the viscosity. Specifically for the local drag on the cylindrical surface, the contribution of the pressure force is always nearly equal to that of the frictional force, as we have seen in Fig. 13. Therefore, the stronger shearing effects in the tip region will lead to the increase of the frictional force and the pressure force, and the drag will increase accordingly.

**Figure 15:**
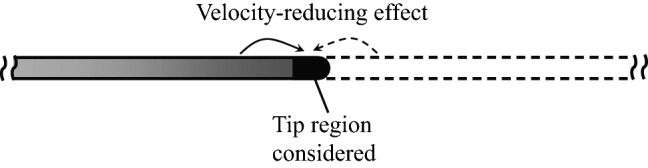
Schematic diagram for the explanation of the increased flow velocity around the tip region of the 3-D single cylinder.

From the above discussion on the single 3-D cylinder, we see that the 3-D effect increases the flow velocity (relative to that of the 2-D case) around the tip region, resulting in larger frictional and pressure forces, hence the larger drag in the region. As for why the flow velocity in the tip region is increased, we give a possible explanation here. Figure 15 shows a schematic diagram of the 3-D single cylinder (the shaded part). Let us consider the tip region of the cylinder. Supposing the cylinder extended beyond the tip to infinity, as shown by the dashed lines in the figure, the ”tip segment” would be like a segment of a 2-D cylinder. From the discussion in the last section, it is known that a 2-D cylinder will reduce the velocity of the flow around it. Therefore, we can assume that the flow around a segment of a 2-D cylinder is affected (velocity reduced) by other segments of the cylinder. In the 2-D case, the flow around the ”tip segment” would be affected by the segments on both sides of it (as indicated by the arrows in Fig. 15). In the 3-D case, however, there are no segments beyond the tip. Thus, the flow velocity around the tip region of the 3-D cylinder is less reduced and hence larger than that around the 2-D cylinder.

**Figure 16:**
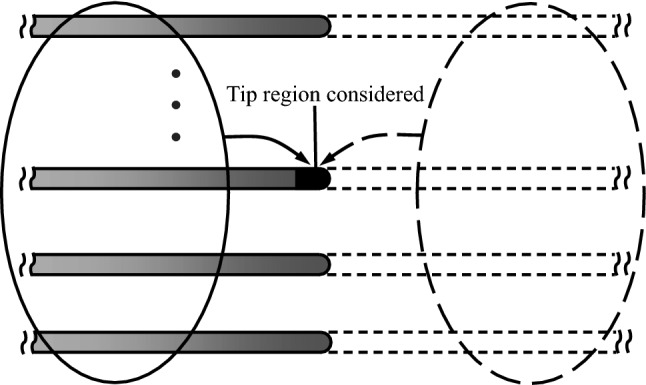
Schematic diagram for the explanation of the stronger 3-D effect in the bristled wing than in the single cylinder.

Now, let us come back to the 3-D bristled wing which consists of a row of 3-D cylinders. As seen in Fig. 9, drag coefficient in the tip region of each cylinder of the wing is increased relative to that of the corresponding cylinder in the 2-D bristled wing. This is in general similar to the case of the single 3-D cylinder that has just been discussed above. But there is a quantitative difference: For the single 3-D cylinder, the length of the tip region affected by the 3-D effect is approximately 1/5 of the span, and the peak value of $C_d$ is about 2.2 times of $C_d$ at the mid-span (see Fig. 12); whereas for a cylinder of the bristled wing, the length of the affected tip region is approximately 1/3 of the span, and the peak value of $C_d$ is more than 3 times of $C_d$ at the mid-span (see Fig. 9). This shows that the ”multi-cylinder wing” (bristled wing) has a stronger 3-D effect than the single cylinder. We can explain this using the sketch in Fig. 16, in which the 3-D bristled wing is represented by parallel shaded cylinders. Let us consider the tip region of a cylinder, say, cylinder No. 3. If the cylinders in the 3-D wing extend beyond their tips to infinity, as shown by the dashed lines in the figure, the ”tip segment” would be like a segment of the corresponding cylinder in a 2-D wing and the flow around it will be affected (velocity reduced) by all the other segments, including not only the segments of its own, but also those of other cylinders. However, for the case of the 3-D wing, there is no velocity-reducing effect from the segments beyond the tips, including not only the segments beyond its own tip, but also those beyond other cylinders’ tips. Therefore, for the tip region of a cylinder in the 3-D bristled wing, the 3-D effect will enhance the flow velocity around it more than the 3-D effect will do to the case of the 3-D single cylinder. Thus we have explained why the 3-D effect of the 3-D bristled wing is stronger than that of a 3-D single cylinder.

The aforementioned mechanism well accounts for the 3-D effect of the finite bristled wing during the translational motion when the incoming flow is uniform and the spanwise drag distribution is caused solely by the finite span. Nonetheless, if the wing performs a rotational motion, the incoming flow will be nonuniform: the closer to the wing tip, the larger the velocity of the incoming flow will be. Although we believe the 3-D effect caused by the finite span will still be limited to the tip region, the drag distribution along the span will be different from what has been shown above. To address this, future work is desired.

### Different aspect ratios

**Figure 17:**
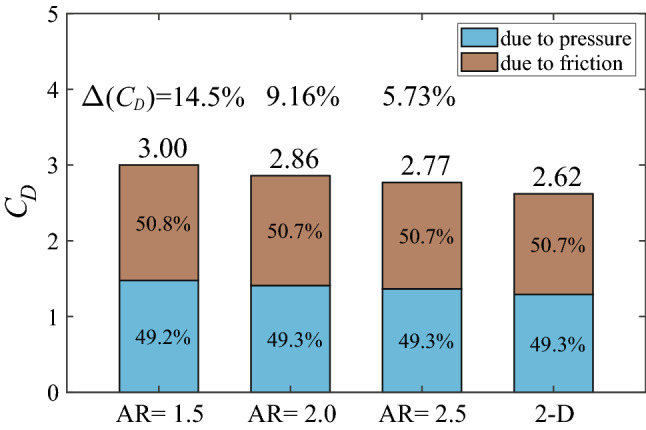
Drag coefficients of the bristled wings with different aspect ratios.

In the previous sections, we considered the case of the wing with AR = 1.5. This aspect ratio is relatively small. It is of interest to explore the effect of increasing the aspect ratio. Here we consider two more bristled wings with larger aspect ratios (AR = 2.0 and 2.5). The computed drag coefficients of the bristled wings are given in Fig. 17 (results of the wing with AR = 1.5 and the 2-D wing are included for comparison). It is seen that when AR increases, the drag coefficient $C_D$ decreases. When AR = 2.0 and 2.5, $\Delta (C_D)$’s are only 9.16% and 5.73%, respectively.

**Figure 18:**
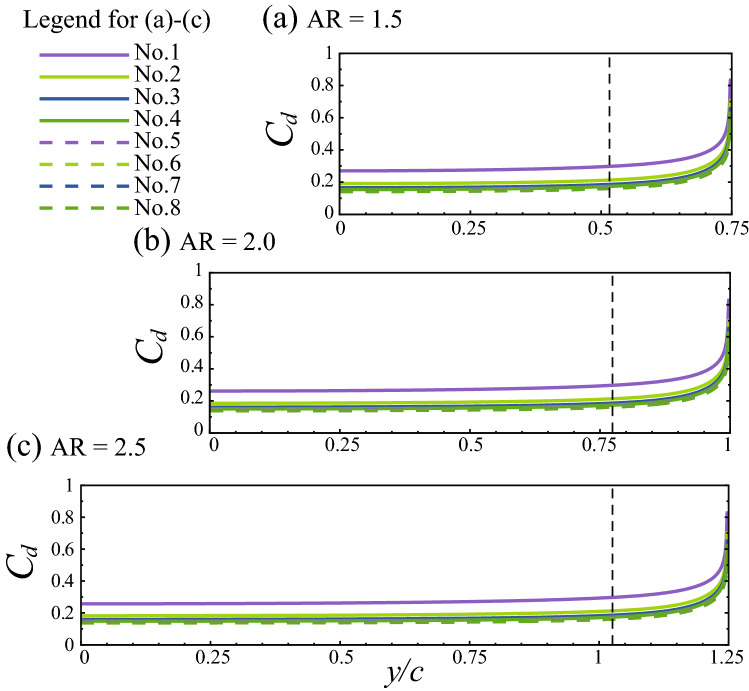
Spanwise distributions of the drag coefficients of each individual cylinder in the 3-D bristled wings with different aspect ratios. The vertical dashed line marks where $C_d$ of the 3-D wing is 10% larger than that of the 2-D wing.

Despite decreasing with the increasing AR, $C_D$ of the 3-D wing is always larger than that of the 2-D wing. In order to identify where the increased $C_D$ comes from, as done previously for the case of AR = 1.5, let us look at the spanwise distribution of the drag coefficient of each individual cylinder in the bristled wings (i.e., $C_d$ varies as a function of *y*). The results (together with the results of AR = 1.5) are given in Fig. 18. From the figure we can see that, similar to the case of AR = 1.5, the distribution curves of cases with larger aspect ratios are also almost horizontal lines that approach the drag coefficients of the corresponding cylinders in the 2-D wing in the inner span and have sharp rises near the tips. Therefore, the increased drag coefficients of the 3-D wings with different aspect ratios are all mainly from the wing tip regions.

From Fig. 18, it can also be seen that for the cases considered, the length of the tip region affected by the 3-D effect almost does not change as the wing length (or, AR) changes. In Fig. 18, the dashed line marks the spanwise location where the sum of ${C_d}$ of every cylinder of the 3-D wing (let us call it ${C_d}$ of the wing) is 10% larger than that of the 2-D wing. The distance between the dashed line and the wing tip could be taken as the length of the tip region affected by the 3-D effect, which is approximately 0.23*c* for the three cases. Moreover, the peak values of ${C_d}$’s of corresponding cylinders in the three cases are almost equal. For the three wings, integrating ${C_d}$ of the wing between the dashed line and the wing tip and non-dimensionalizing the result with the distance between the dashed line and the wing tip gives 3.718, 3.724, and 3.724, respectively. This means the drag coefficient of the tip region affected by the 3-D effect hardly varies with the aspect ratio. Therefore, the 3-D effect in the tip region of the 3-D wing causes the same amount of total drag increment over the 2-D wing. This is a very interesting result.

## Conclusion

Through the analysis of the detailed flow field and the aerodynamic force of the 3-D bristled wing model at the fixed chord-based *Re* of 10, the following findings are obtained: (i) The drag coefficient of the 3-D bristled wing decreases as the aspect ratio increases and is always larger than that of the corresponding 2-D bristled wing (by 14.5%, 9.16%, and 5.73% when AR = 1.5, 2.0, and 2.5, respectively). This is contrary to the 3-D effect at high *Re* (e.g., the drag coefficient of the 3-D cylinder increases as the aspect ratio increases and is always smaller than that of the corresponding 2-D cylinder at high *Re*). The increased drag coefficient of the 3-D bristled wing over the 2-D wing comes from the wing tip regions. In the wing tip region, the flow velocity around the cylinder (bristle) increases dramatically due to the vanishment of the cylinder surface beyond the tip. (ii) For the wings with different aspect ratios considered in this study (AR = 1.5, 2.0, and 2.5), the lengths of the tip regions affected by the 3-D effect are all about 0.23*c*, and the peak values of the drag coefficients are approximately equal, and so are the drag increments in the tip region (caused by the 3-D effect). That is, for the bristled wings considered, the 3-D effect in the tip region almost does not vary with the aspect ratio.

Because the 3-D bristled wing model and the motion of the wing in this work are quite basic, future work could probe deeper into more sophisticated models and motions (e.g., how the radial arrangement of the bristles will affect the drag of the wing; what the drag distribution of the wing will be like during rotation, or, a real flapping motion). Understanding why the bristled wing only appears in the smallest insects and the potential benefits of this kind of configuration will be helpful to the design of the micro air vehicles.

## Data Availability

The datasets used and analysed during the current study are available from the corresponding author on reasonable request.
